# Lambda-Restricted Crystal-Storing Histiocytosis of Stomach: A Case Report and Review of Literature

**DOI:** 10.7759/cureus.15009

**Published:** 2021-05-13

**Authors:** Nalini Bansal, Pankaj Puri, Nishant Nagpal, Rahul Naithani, Rahul Gupta

**Affiliations:** 1 Histopathology, Fortis Escorts Heart Institute, New Delhi, IND; 2 Gastroenterology and Hepatology, Fortis Escorts Heart Institute, New Delhi, IND; 3 Hematology and Oncology, Max Hospital Saket, New Delhi, IND; 4 Gastrointestinal Surgery, Synergy Institute of Medical Sciences, Dehradun, IND

**Keywords:** histiocytosis, stomach ulcers, non-hodgkins lymphoma, linear crystals, monoclonal immunoglobulins

## Abstract

Crystal-storing histiocytosis (CSH) is a rare tissue phenomenon that is usually associated with lympho-proliferative diseases. The disease is characterized by prominent collections of macrophages with abundant eosinophilic cytoplasm and fibrillary cytoplasmic inclusions. The inclusions appear as linear crystals within the macrophages which are usually kappa restricted. The disease usually involves lungs, lymph nodes, bone marrow, thymus and spleen with rare involvement of the gastrointestinal tract. We report a rare case of lambda-restricted CSH of the stomach. The diagnosis of CSH triggered further hematological evaluation. The patient was later diagnosed to have diffuse large B-cell lymphoma involving lymph nodes and bone marrow. He received chemotherapy for the same and is on regular follow up. The index case highlights the need to identify CSH of stomach prompting evaluation for hematological malignancies and to increase its awareness among clinicians and pathologists.

## Introduction

Crystal-storing histiocytosis (CSH) is a rare disorder of histiocytic proliferation that usually occurs in patients with underlying hematological malignancies. Histiocytic proliferation shows a unique accumulation of crystals of monoclonal immunoglobulins. The knowledge of this entity is crucial to avoid misdiagnosis of plasma cell neoplasms as immunoglobulin deposition is a usual feature of the latter. The disease usually involves the lung, lymph node, bone marrow, thymus and spleen with rare involvement of the gastrointestinal tract. Involvement of the stomach is rarely reported in the English literature [1-9]. We report the case of lambda-restricted gastric CSH, which later uncovered an underlying diffuse large B-cell lymphoma (DLBCL) involving multiple lymph nodes and bone marrow.

## Case presentation

An 86-year-old male presented with complaints of two episodes of hematemesis and abdominal pain for one day. On clinical examination, the patient was hemodynamically stable with no jaundice, clubbing, cyanosis or edema. Abdominal examination revealed mild tenderness in the epigastric region. There were no neurological deficits. Laboratory investigations revealed hemoglobin 12.0 g/dL (normal range: 13.0-17.0 g/dL), white blood cell count 8.18 thou/uL (4.0-10.0 thou/uL), platelet count 174 (150-410 thou/uL), hematocrit 35.5 (40%- 50%) with unremarkable differential count. The coagulation profile, liver and renal function tests were within the normal range. Serum lactate dehydrogenase level was elevated [473 U/L (normal range: 135-225 U/L)].

**Figure 1:**
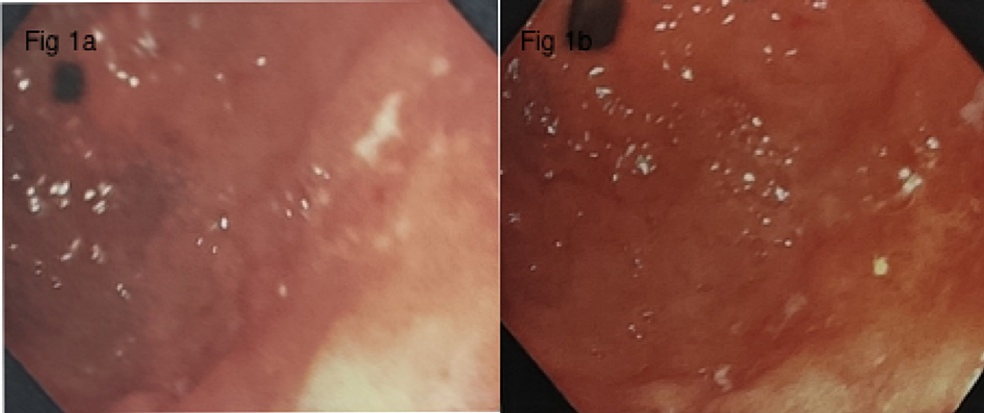
Upper gastrointestinal endoscopy at admission (a) and after two days (b) showing the gastric ulcer located in the antrum.

In view of gastrointestinal bleed, an emergency upper gastrointestinal endoscopy (UGIE) was done that showed Schatzki ring, Forrest IIB gastric ulcer in the antrum along the lesser curvature with adherent clot. Inj. Adrenaline (1:10,000) was injected in all four quadrants of the ulcer. He was started on intravenous proton pump inhibitors. Repeat UGIE done two days later showed large ulcer seen in antrum at the incisura, clean based (Forrest III) with surrounding induration (Figures 1a, 1b). Multiple biopsies were taken. The rapid urease test for Helicobacter pylori was positive. Another small ulcer was seen in the antrum with patchy erythema and induration.

**Figure 2:**
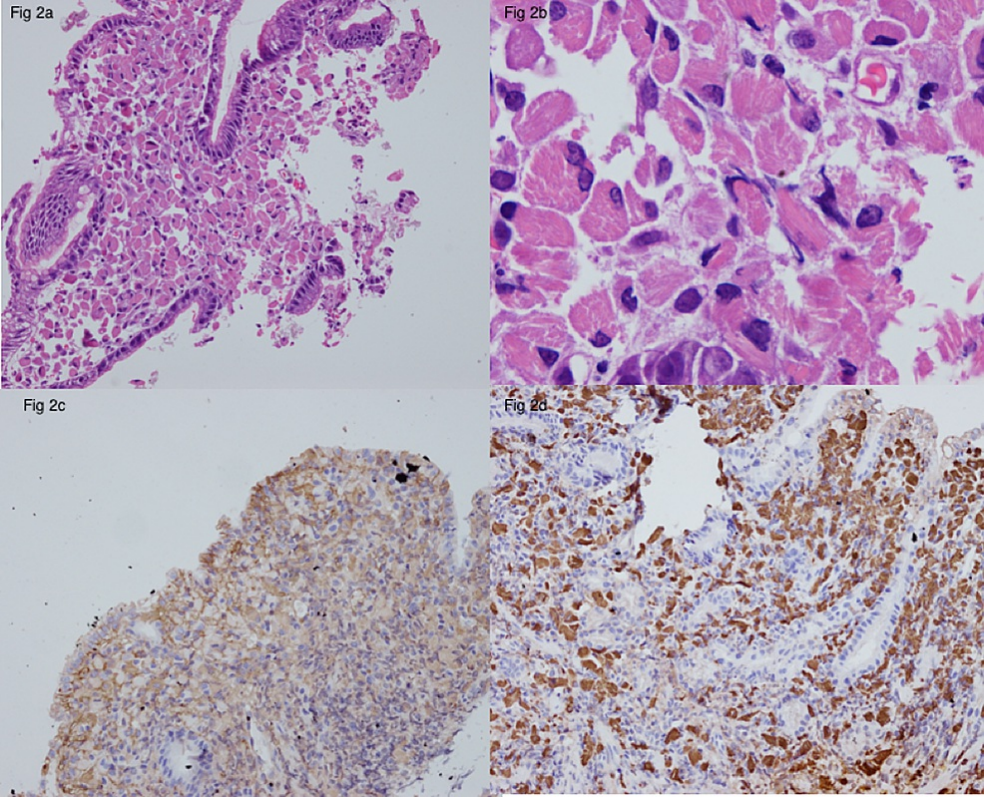
Microscopic examination of the gastric biopsy showing histiocyte aggregates in lamina propria (a) and crystals within histiocytes (b). Immunohistochemistry showing kappa (c) and lambda (d) within the histiocytes.

The patient improved symptomatically. He was started on Helicobacter pylori eradication therapy (sequential therapy). The gastric biopsy tissue revealed the presence of large cells with abundant eosinophilic cytoplasm and the presence of linear crystals. Immunohistochemistry (IHC) performed for plasma cells (CD 138) was negative, and the cytoplasmic content was strongly lambda positive with weak kappa staining (Figures 2a-2d). The large cells were CD 68 positive and negative for pancytokeratin (PanCK), leukocyte common antigen (LCA) and CD 20. The final diagnosis of CSH was rendered.

In view of CSH, the patient underwent further evaluation for the myeloma panel which was negative. The patient underwent a PET scan, which showed fluorodeoxyglucose (FDG) avid lesions in multiple lymph nodes and spleen. He later underwent trucut biopsy from the left cervical lymph node. The biopsy tissue showed features of DLBCL. In view of lymphoma, he also underwent bone marrow aspiration and biopsy, which showed marrow infiltration by B-cell Non-Hodgkin’s lymphoma. As the performance status of the patient was good, he was started on prednisolone keeping his age in mind. He tolerated the chemotherapy well and is now on regular follow up for one year.

## Discussion

CSH is a rare disease composed of histiocytes with an abnormal intra-lysosomal accumulation of immunoglobulin as crystals [1]. The largest review of CSH involving 80 patients by Dogan et al. found an association with lymphoproliferative plasma cell disorder in 90% cases [10]. The associated lymphomas were B-lymphoproliferative disorders comprising marginal zone lymphoma with plasmacytic differentiation, lymphoplasmacytic lymphoma, monoclonal gammopathy of uncertain significance and plasma cell neoplasms. Most cases of CSH were identified in the head and neck (35%), followed by lung and pleura, bone marrow, kidney, lymph nodes, skin and others. The most common site of CSH in the head and neck region was the eye/orbit [10].

The median age of the patients is 60 years (range: 17-81 years) [1-9]. The male to female ratio is nearly 1:1. The disease can occur in a localized or generalized form. Localized disease is confined to a single organ or site seen in nearly 58% cases compared to generalized form, which involves around 42% cases [10]. The clinical features depend on the site of involvement. Histology in most cases shows the presence of histiocytes containing eosinophilic inclusions within the cytoplasm. The eosinophilic inclusions within the cytoplasm appear as non-refractile, nonpolarizable linear crystals [1]. The inclusions are mainly phagocytized excessive light chains that accumulate within the lysosomes of histiocytes. The light chains can be polyclonal or monoclonal. Monoclonal light chains are typically kappa isotype and rarely of lambda isotype. In the present case, the accumulated light chains showed lambda restriction.

The histiocytes in CSH need to be differentiated from Russel body gastritis. In Russel body gastritis, the cells accumulating the immunoglobulin are plasma cells and not histiocytes. Another feature that differentiates the two disorders are the nature of immunoglobulin deposits, which appear as globular deposits within the cell in Russel body gastritis as opposed to linear crystals in CSH [11]. There are few reported cases of linear crystal deposits within histiocytes other than immunoglobulins such as cystine deposits, Charlot Layden crystals and clofazimine-induced crystals [12-14]. The identification of CSH prompts further evaluation for hematological disorders. Treatment and prognosis are varied according to the underlying disease.

Though CSH has been reported from various sites within the body, involvement of the stomach is very rare. The clinicopathological features of the reported cases have been presented in Table 1 [1-9]. CSH localized to the stomach was seen in 85% cases (11/13 cases). Cases of gastric CSH has been associated with lymphoproliferative disorders in 66% cases inclusive of monoclonal B-cell lymphoproliferative disorders (multiple myeloma, lymphoplasmacytic lymphoma, extranodal marginal zone lymphoma and mantle cell lymphoma). DLBCL was found to be associated in only one case report as was seen in our case. Monoclonality was identified in 66% cases on IHC for kappa and lambda light chains with 87% cases showing kappa restriction. Lambda restriction was noted in only one case. In the index case, the histiocytes showed lambda restriction. Cases of CSH included are only those cases where histiocytes show crystal accumulation. Cases where plasma cells showed similar crystal were disregarded in the study as they can now be better designated as Russell body gastritis.

**Table 1:** A brief literature review of reported cases of crystal-storing histiocytosis of the stomach. CSH – crystal-storing histiocytes, DLBCL – diffuse large B-cell lymphoma, EUS – endoscopic ultrasound, F - female, GERD – gastroesophageal reflux disease, HE – hematoxylin and eosin, IHC - immunohistochemistry, M - male, MALT – mucosa-associated lymphoid tissue, MCL – mantle cell lymphoma, ND – not described

S.No	Authors	Year	Age/ sex	Site of involvement	Associated morbidity	Clinical features	Endoscopic features	Immunoglobin expression	IHC pattern of cells	Follow up
1	Jones et al. [2]	1999	36/F	Stomach, bone marrow, thymus	Childhood lupus hypergammaglobulinemia thymic lymphoma	Epigastric pain	Submucosal lesion. On EUS a 2 cm whitish patch on the greater curvature of the stomach	Polyclonal, IgA	Histiocytes	Persistent disease
2	Joo et al. [3]	2007	56/F	Localized	Helicobacter pylori infection	Dyspepsia and epigastric pain for 2 weeks	Chronic gastritis with three polyps. The polyps were located in the angle, anterior wall of the antrum and distal antrum along the greater curvature, measuring 30 mm, 15 mm and 20 mm, respectively	Polyclonal	Histiocytes and plasma cells-CD79a, CD138 (syndecan-1) and CD68	Reactive CSH, ND
3	Yano et al. [4]	2013	55/F	Localized	Sjogren’s syndrome and Helicobacter pylori gastritis	No symptoms. Incidentally detected during endoscopic screening	Flat, whitish, finely granular membrane with a diameter of 10 mm was seen in the greater curvature of the gastric body	Polyclonal, kappa and lambda	Histiocytes-CD68, CD163, IgG, IgA, kappa, and lambda	Endoscopic submucosal dissection was done with no recurrence at four years.
4	Vaid et al. [5]	2014	Elderly male	Localized	B-cell Lymphoma	Long-standing GERD	Whitish patch	Plasma cells were kappa-restricted on ISH. IHC stains not done for histiocytes	Histiocytes on HE. No IHC done.	On follow up with hemato-oncologist
5	Kangal-Shamanna et al. [1]	2016	43/M	Stomach, esophagus	MALT lymphoma	Abdominal cramps, rectal bleeding; stomach nodules on EGD	ND	Monoclonal, kappa	Histiocytes, IgA, kappa	Alive at 36 months
6	Kangal-Shamanna et al. [1]	2016	51/M	Stomach, bone marrow	Plasma cell myeloma	Persistent progressive abdominal pain, fatigue	ND	Polyclonal	Histiocytes-CD 163, CD68 and IgG, Weak blush of kappa nd lambda	Alive at 26 months
7	Isono et al. [6]	2016	54/M	Localized	None	None, lesion was detected on abdominal screening	25-mm brown region in the angular section of the greater curvature of the stomach	Monoclonal IgG Kappa	Histiocytes-CD 68	6 years survival with brain stem infarction
8	Arnold et al. [7]	2018-4 patients	56-82 y, males (n = 2) and females (n = 2)	Localized	Mantle cell lymphoma (n = 1), DLBCL (n = 1), MALT lymphoma (n = 2)	Worsening gastroesophageal reflux, abdominal pain, melena, and diarrhea	Diffuse nodularity and white discoloration of body and antrum (n = 1), patchy nodularity (n = 1), and malignant-appearing fundic mass with lymphadenopathy (n = 2)	All monoclonal, kappa restricted in three and lambda restricted in one	Histiocytes (n = 2), Histiocytes, kappa restricted (n = 2)	All four cases associated with lymphoproliferative disorder. Two also had H pylori gastritis. MCL died at week 16, DLBCL died at week 133, Remaining two alive
9	Fujita et al. [8]	2018	72/F	Localized	None	Chronic gastritis	Diffuse granular mucosa from the gastric fundus to the body, The mucosa of the antrum exhibited no remarkable changes except slight swelling. Magnification endoscopy with narrow-band imaging revealed expanded inter-crypt spaces with abundant capillaries in the granular mucosa.	Monoclonal IgA Kappa	Histiocytes-CD 68, IgA, Kappa	No underlying disorder on five-year follow up.
10	Joo et al. [9]	2020	56/M	Localized to stomach	MALT lymphoma	Hematemesis and malena	1 cm gastric ulcer with eroded vessel located in the body of the stomach	Monoclonal, kappa	Histiocytes-CD 68, kappa	No treatment received
11	Present case	2021	86/M	Localized	DLBCL	Abdominal pain, hematemesis	Gastric ulcer – Forrest IIB in the antrum along lesser curvature	Monoclonal, lambda	Histiocytes-CD 68, Lambda	Received chemotherapy

## Conclusions

Gastric CSH is a very rare pathological finding. It should prompt further hematological work up as most of the cases are associated with lymphoproliferative disorders. High clinical suspicion and increased awareness among clinicians and pathologists are required to avoid delay in the diagnosis and treatment of underlying hematological disorders.
